# Close contact interferon-gamma response to the new PstS1_(285–374)_:CPF10: a preliminary 1-year follow-up study

**DOI:** 10.1186/s13104-016-2360-4

**Published:** 2017-01-23

**Authors:** Leonardo Silva de Araujo, Nidai de Bárbara Moreira da Silva Lins, Janaina Aparecida Medeiros Leung, Fernanda Carvalho Queiroz Mello, Maria Helena Féres Saad

**Affiliations:** 1 Laboratory of Cellular Microbiology, Oswaldo Cruz Institute, Fiocruz, Avenida Brasil, 4365, Rio de Janeiro, RJ 20045-360 Brazil; 2 Federal University of Rio de Janeiro, Helio Fraga Filho Hospital, Professor Rodolpho Paulo Rocco Street, 255, 1st Floor, Ilha do Fundão, Rio de Janeiro, RJ 21941-913 Brazil

**Keywords:** *M. tuberculosis*, Tuberculosis, LTBI, Interferon-gamma, Follow up

## Abstract

**Background:**

The available diagnostic tools for latent tuberculosis (TB) infection (LTBI) via interferon-gamma (IFN-g) release assays (IGRA) are based on ESAT6:CFP10 stimulation. However, the mycobacterial antigen PstS1 is also highly immunogenic and some of its fragments, such as PstS1_(285–374)_, have shown higher immunoreactivity in LTBI than in active TB. PstS1_(285–374)_, therefore, could increase the accuracy of the existing IGRA to detect LTBI. Thus, a new chimeric protein has recently been developed (PstS1_(285–374)_:CFP10) showing potential for LTBI screening of recent close contacts (rCt) exposed to *Mycobacterium tuberculosis*. The aim of this study was to analyze the PstS1_(285–374)_:CFP10 longitudinal IFN-g profile in comparison to ESAT6:CFP10 and full PstS1/CFP10 stimulation in a rCt cohort and correlate the responses to these in-house IGRA with any clinical changes/interventions that might occur.

**Methods:**

A free-of-cost, one-year follow up was offered to 120 rCt recruited in Rio de Janeiro, RJ, Brazil. Whole blood short-term (WBA), long-term stimulation (LSA) assays, and the tuberculin skin test (TST) were performed during follow up.

**Results:**

Among the enrolled rCt, 44.2% (53/120) returned for re-evaluation and the control group (TST negative, n = 17) showed low IFN-g reactivity to all antigen stimulations during the entire follow up, except for one participant who had shown radiological evidence of past TB/LTBI. Both incident cases were detected by IGRA-PstS1_(285–374)_:CFP10 during LTBI and after disease progression. Moreover, subsequent to the prophylactic treatment for LTBI (tLTBI), a significant regression in the LSA response was predominantly observed through stimulation of the new chimeric protein (8/10, 80%) followed by ESAT6:CFP10 (5/10, 50%) and PstS1/CFP10 (4/10, 40%). No clinical or epidemiological characteristics were exclusively shared among IGRA convertors.

**Conclusion:**

It was demonstrated that the TST negative rCt without radiological evidence of LTBI/TB did not develop an IGRA-PstS1_(285–374)_:CFP10 response during the one-year follow up. Moreover, all incident cases were detected by our new IGRA; and a significant decrement of LSA-PstS1_(285–374)_:CFP10 reactivity post-prophylactic tLTBI was found. To our knowledge, this is the first study to monitor changes in the immune response profile of IGRA-PstS1_(285–374)_:CFP10 among rCt during a consecutive one-year period, thus providing additional evidence of its potential in the detection of LTBI.

**Electronic supplementary material:**

The online version of this article (doi:10.1186/s13104-016-2360-4) contains supplementary material, which is available to authorized users.

## Background

Despite the availability of efficient antibiotic treatments, the control of tuberculosis (TB) remains a challenge worldwide [1–3]. After an initial *Mycobacterium tuberculosis (Mtb)* infection, approximately 10% will develop TB. The great majority (roughly 90%), however, is expected to control the bacterial replication and present latent infection (LTBI) while under the constant risk of developing the active form [4].

In active disease, clinical manifestations may help diagnose pulmonary TB [5]. In contrast, LTBI is asymptomatic and the lack of adequate diagnostic methods to detect latent infection tend to hamper attempts at preventing new incident cases even though the isoniazid preventive treatment for LTBI (tLTBI) has been found to reduce the risk of developing TB by as much as 90% [2, 6].

For over a century, LTBI diagnoses have relied on the relatively simple, low-cost, tuberculin skin test (TST) [7]. However, false-positive results may occur in the TST due, in large part, to a previous exposure to environmental non-tuberculous mycobacteria [8]. TST also includes the boosting phenomenon and the need for a reading visit [9]. After development of the commercial interferon-gamma (IFN-γ) release assay (IGRA), a new tool emerged that measures the host T helper 1 (Th1) IFN-γ response by priming blood cells with antigens encoded by the *Mtb* region of difference 1 (RD1), the ESAT6, and the CFP10 [2]. Notwithstanding the evidence afforded by longitudinal studies suggesting that, among high-risk populations, TST is more sensitive [10] and cost-effective [2], there is a global tendency, especially in high-income countries, to replace TST by a single-step commercial IGRA-RD1 or simply apply it after a prior TST screening [11]. But, these approaches would necessarily increase the financial burden of global TB control programs, primarily in low- and middle-income countries [1, 12]. Hence, the need for new, more cost-effective, and reliable tools/antigens that could safely substitute or add sensitivity to the LTBI biomarkers currently in use continues to exist.

A new PstS1_(285–374)_:CFP10 fusion protein has recently been designed by our group. Among LTBI, in comparison to the traditional ESAT6:CFP10, our new chimeric protein has demonstrated similar immunoreactivity while slightly increasing the detection of TST positive (TST^pos^) rCt by both WBA and LSA [13]. This new protein was also able to identify an incident case that progressed from LTBI to active pulmonary TB within a short period in the absence of a response to LSA-ESAT6:CFP10 [13]. As both TST and IGRA RD1-based have their limitations, continued surveillance of host immunomodulatory changes for a specific length of time, and especially after recent *Mtb* exposure, may result in revealing new antigens that could potentially be used to either compose new diagnostic tests or augment the effectiveness of current ones. In this connection, the aim of the present study was to determine the longitudinal IFN-γ profile via whole blood short-term (WBA) and peripheral blood mononuclear cell (PBMC) long-term stimulation (LSA) assays in a cohort of recent close contacts (rCt) exposed to a pulmonary TB index case (IC). All participants were recruited from the general patient population seeking treatment at the public health care facilities located in the City of Rio de Janeiro. The present study correlated the modulations detected in WBA and LSA responses under PstS1_(285–374)_:CFP10, PstS1/CFP10, and ESAT6:CFP10 stimuli resulting from clinical changes and antibiotic interventions.

## Methods

### Study samples

The present investigation was carried out on samples previously collected by the TB Control Program at the Clementino Fraga Filho University Hospital in Rio de Janeiro, RJ, Brazil [13]. All rCt recruited between March 2010 and August 2012 were >17 years of age and tested negatively for the human immunodeficiency virus (HIV). Upon obtaining informed oral and written consent, blood samples were collected prior to administering the Mantoux TST [13].

According to Brazilian Ministry of Health recommendations [14], all close contacts showing a TST^pos^ (cut-off ≥5 mm) with no indication of disease were considered LTBI or as having pulmonary TB in light of their respective microbiological, radiological, and clinical findings and were subsequently offered specific free-of-cost treatment.

### Prospective analysis

The TB Control Program offered clinical follow up to all rCt and the TST negative cases were retested at four-month intervals and after the twelfth month, while TST positive subjects were just treated and clinically followed. In a previous study [13] by our group analyzing the potential use of PstS1 _(285–374)_:CFP10 as a new LTBI biomarker, blood samples were taken upon enrollment (T_0_). In the present study, the rCt were followed up longitudinally for a year (T_1_), except for the TST convertors and incident cases, from whom blood was collected upon a skin test conversion or TB diagnosis. For the in vivo TST, a conversion was classified as an augmentation of ≥10 mm in comparison to a previously reported test. Conversely, for the in vitro WBA and LSA, a conversion**/**regression response was defined as a respective augmentation or decrement that crosses the cut-off points in the follow-up tests in comparison to baseline reactivity.

### *Mycobacterium tuberculosis* antigens

The *Mtb* recombinant antigens ESAT6:CFP10 and PstS1/CFP10 were kindly provided by Dr. Tom Ottenhoff (Leiden University Medical Centre, The Netherlands) and Dr. Mahavir Sigh (Helmholtz Centre for Infection Research, Germany), respectively. The chimeric protein PstS1_(285–374)_:CFP10 was produced in our laboratory, as previously described [13]. In brief, sequences of the selected genes were amplified from *M. tuberculosis* H37Rv genomic DNA using gene-specific primers. Each fragment was then cloned, fused, and transformed for recombinant expression in *Escherichia coli*.

### Whole blood (WBA) and peripheral blood mononuclear cell (PBMC) long-term stimulation assays (LSA)

As previously described [13], heparinized venous blood was collected and separated for WBA (1 mL per well for 22 h) or for PBMC isolation followed by LSA (1 × 10^5^ cells/well, 5 days) in the presence or absence of Concanavalin-A/antigens. The culture supernatants were collected and stored at −20 °C until tested.

The commercial ELISA Duo-Set-IFN-γ kit (R&D, USA) was used to evaluate IFN-γ secretion following the manufacturer’s recommendations. After subtraction of the appropriate unstimulated control, the results were expressed in picograms/milliliters (pg/mL). All cut-off points were previously standardized (**WBA**: ESAT6:CFP10 = 10 pg/mL; PstS1/CFP10 = 10.5 pg/mL; PstS1_(285–374)_:CFP10 = 29.5 pg/mL; **LSA**: 100 pg/mL each) [13].

### Data analysis

Prism5 (GraphPad Software, USA) and SPSS 17.0 (IBM, USA) were used to generate plots and for statistical analyses. The Mann–Whitney and Kruskal–Wallis nonparametric tests were utilized to analyze significant differences between 2 or >2 groups, respectively. The Wilcoxon Signed Rank Test was adopted for longitudinal analyses. P values of <0.05 were considered significant.

## Results

### Study participants

**Fig. 1:**
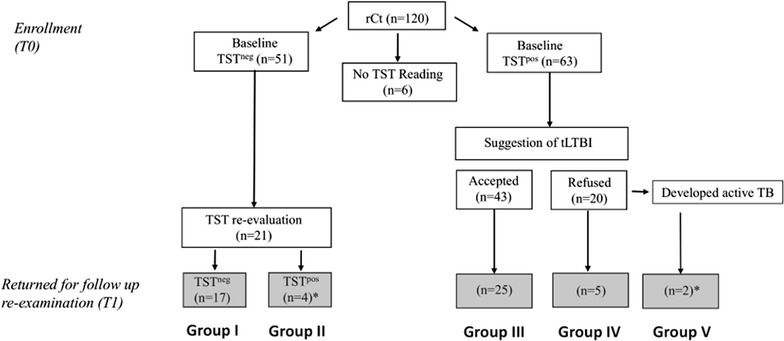
Baseline of study groups (T_0_) and follow-up (T_1_) characteristics. Recent close contact (rCt) screening via tuberculin skin test (TST), at T_0_, and clinical outcomes after 1-year follow up (T_1_) resulting in five different groups. All rCt-TST^pos^ (≥5 mm) were offered free-of-cost treatment for latent tuberculosis infection (tLBTI). A blood specimen was collected upon TST conversion or an active TB diagnosis

According to Brazilian Ministry of Health guidelines, a clinical evaluation/follow up and TB prophylaxis are not mandatory after reported exposure to *Mtb*. Of all enrolled rCt, 44.2% (53/120) returned for the 1-year re-evaluation. They were then organized into study groups according to their baseline TST and clinical outcomes, as follows: Group I, TST negative (TST^neg^: WBA, n = 10; LSA, n = 17); Group II, TST conversion (WBA, n = 2; LSA, n = 4); Group III, TST^pos^ submitted to tLTBI (WBA, n = 17; LSA, n = 25); Group IV, TST^pos^ untreated (WBA, n = 4; LSA, n = 5); and Group V: TB incident cases (WBA, n = 2; LSA, n = 2) (Fig. 1).

As shown in Table 1, while nearly half (49.1%) of the enrolled rCt inhabited the same dwelling as their IC, the proportions of gender and BCG vaccination status were slightly asymmetrical (58.5% was female and 62.3% had a BCG vaccination scar). The mean ages were similar among all groups, varying between 43.5 (±14.4) and 46.4 (±18.5) years of age (p ≥ 0.051), except for **Group V** (61.5 ± 3.5 years, p = 0.023). As expected, there was a statistical difference (p ≤ 0.016) when comparing mean skin test induration between TST^pos^ (Group III or IV) and TST^neg^ (Group I or II) rCt.

**Table 1:** Baseline demographic characteristics of the study population^a^

Baseline characteristics	Number of participants (%)					
	All 53 (100)	Group I 17 (32.1)	Group II 4 (7.5)	Group III 25 (47.2)	Group IV 5 (9.4)	Group V 2 (3.8)
BCG scar	33 (62.3)	9 (52.9)	4 (100)	18 (72)	2 (40)	0 (0)
Females	31 (58.5)	8 (47.1)	3 (75)	16 (64)	3 (60)	1 (50)
Household contact	26 (49.1)	5 (29)	2 (50)	10 (40)	2 (40)	2 (100)
Mean (±SD) age, years	45.1 (±13.2)	43.9 (±13.0)	43.5 (±14.4)	44.5 (±12.6)	46.4 (±18.5)	61.5 (±3.5)^¥^
Mean (±SD) TST induration, mm	7.2 (±6.1)	0.4 (±1.2)	1.8 (±2.1)	11.8 (±3.7)*^¥^	9.8 (± 3.6)*^¥^	12.5 (±3.5)*

Group I TST^neg^, Group II TST convertors, Group III TST^pos^ submitted to treatment for latent tuberculosis infection (tLTBI), Group IV TST^pos^ not submitted to the tLTBI, and Group V developed active pulmonary TB. Mann–Whitney test was used to generate *p* values

^a^ *BCG* Bacille Calmette Guérin, *mm* millimeters, *TST* tuberculin skin test, *SD* standard deviation

The symbols * and ^¥^ were used to identify p values <0.05 comparing to Group I or II, respectively

### IGRA reactivity

Due to ethical limitations, the adequate amount of blood for both assays was unavailable for some rCt, thus justifying the discrepancies between the sample sizes of WBA and LSA.

**Fig. 2:**
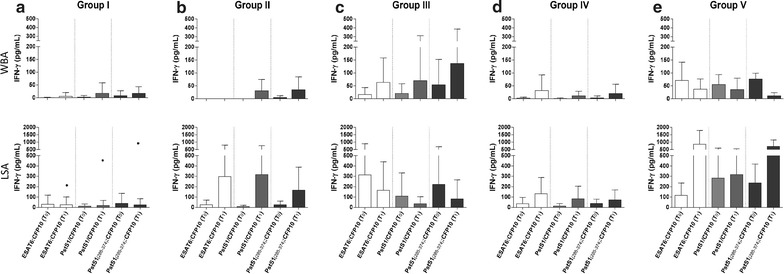
Mean, standard deviation (SD) of interferon-gamma responses at enrollment (T_0_) and after follow up (T_1_). Longitudinal analysis via Wilcoxon signed rank test of mean interferon-gamma levels after short- (WBA) and/or long-term (LSA) stimulation assays priming blood cells from recent close contacts with mycobacterial antigens [ESAT6:CFP10, PstS1/CFP10, and PstS1(285–374):CFP10] upon enrollment (T_0_) and after one-year follow up (T_1_). In some special cases, a blood specimen was collected upon TST conversion or an active TB diagnosis. All individuals in the study were stratified according to their initial (T_0_) tuberculin skin test (TST) response and T_1_ outcome, as follows: **a** Group I TST^neg^ (n, WBA = 10, LSA = 17); **b** Group II TST convertors (n, WBA = 2, LSA = 4); **c** Group III TST^pos^ treated for latent tuberculosis infection (tLTBI) (n, WBA = 17, LSA = 25);** d** Group IV TST^pos^ not treated for tLTBI (n, WBA = 4, LSA = 5); and **e** Group V TB incident cases (n = 2). *Short bars* standard deviation

With the exception of one outlier at T_1_ (Black cirle, Fig. 2a) who had a TB compatible chest x-ray since 1978 with no other clinical manifestations, the mean amount of IFN-γ detected in Group I was similarly low at the two time-points for both IGRAs (p ≥ 0.109). In contrast, the PBMCs of participants with a TST conversion (Group II, Fig. 2b) or untreated TST^pos^ (Group IV, Fig. 2d) demonstrated a slight-to-moderate increase in their mean IGRA responses (p ≥ 0.109).

An exclusive, antagonic IFN-γ modulation during short-and long-term incubation periods was noticed in Groups III (tLTBI) and V (TB). While the mean LSA response dropped post-tLTBI (Fig. 2c, p ≥ 0.071), the WBA response increased (p ≥ 0.084). Interestingly, the Group V response was the opposite: the mean WBA response dropped and LSA increased along the follow-up period, principally in relation to ESAT6:CFP10 and PstS1_(285–374)_:CFP10 (Fig. 2e, p ≥ 0.18).

Despite the tendencies regarding IFN-γ modulation, no statistically significant changes were identified when comparing paired IFN-γ mean ± standard deviation (SD) reactivity to any antigenic stimulation (Fig. 2, p ≥ 0.071). The same was observed for concanavalin A (p ≥ 0.087) in both the **WBA** (mean ± SD, pg/mL, T_0_ = 74.1 ± 99.0 and T_1_ = 84.2 ± 97.6; n = 35) and **LSA** (mean ± SD, pg/mL: T_0_ = 2268.9 ± 1831.1 and T_1_ = 1704.9 ± 874.8; n = 55). All the mean IFN-γ responses are shown in Additional file 1: Table S1.

**Fig. 3:**
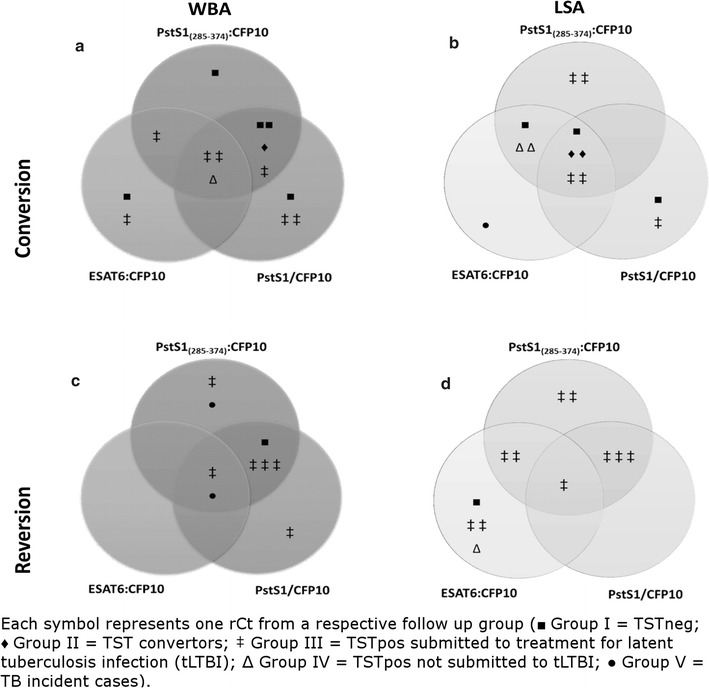
Venn diagrams for the follow up of in-house interferon gamma release assay results. Distribution of interferon-gamma response conversion (**a**, **b**) or regression (**c**, **d**) at follow-up whole-blood (WBA) and long-term (LSA) stimulation assays. Recent close contact’s blood specimens were primed with *Mycobacterium tuberculosis* antigens (ESAT6:CFP10, PstS1/CFP10, and PstS1_(285–374)_:CFP10). Each symbol represents one rCt from the respective follow-up group (*Black square Group I* TST^neg^; *Black diamond Group II* TST convertors; *Double dagger Group III* TST^pos^ treated for latent tuberculosis infection (tLTBI); *White triangle Group IV* TST^pos^ not tLTBI; *Black circle Group V* TB incident cases)

Interferon-gamma response regressions or conversions were observed in some rCt. Either WBA or LSA conversions occurred in all individuals regardless of group (**WBA-convertors:** Group I: 5/14 [35.7%]; Group II: 1/14 [7.1%]; Group III: 7/14 [50%]; Group IV: 1/14 [7.1%]. **LSA-convertors:** Group I: 3/13 [23.1%]; Group II: 2/13 [15.4%]; Group III: 5/13 [38.5%]; Group IV: 2/13 [15.4%]; and Group V: 1/13 [7.6%]) as well as to all antigens (Fig. 3 a, b). However, no clinical or epidemiological characteristics were exclusively shared among the IGRA convertors.

Conversely, regression from a previously positive IFN-γ response was mostly seen in Group III whatever the stimulus (Group III regressors/Total regressors: **WBA**: 6/9, 66.7%; **LSA**: 10/12, 83.3%), but principally to the new PstS1_(285–374)_:CFP10 antigen (**WBA**: ESAT6:CFP10: 2/9, 22.2%; PstS1/CFP10: 7/9, 77.8%; PstS1_(285–374)_:CFP10: 8/9, 88.9%. **LSA**: PstS1/CFP10: 4/12, 33.3%; ESAT6:CFP10: 7/12, 58.3%; PstS1_(285–374)_:CFP10: 8/12, 66.6%) (Fig. 3 c, d).

### Effects of tLTBI on in-house IGRA responses

**Fig. 4:**
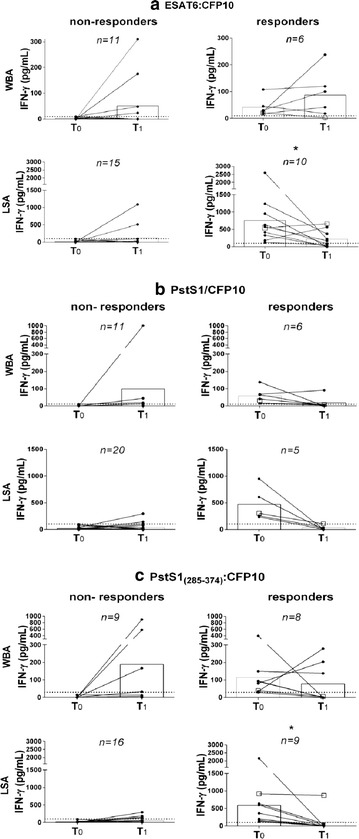
Longitudinal interferon-gamma responses post-prophylactic treatment for latent tuberculosis infection. Longitudinal analysis upon enrollment (T_0_) and after 1-year follow up (T_1_) of mean interferon-gamma levels via short- (WBA) and long-term (LSA) stimulation assays with blood specimens from recent close contacts of TST^pos^ treated for latent tuberculosis infection (*Black circle*) and stratified according to their baseline interferon-gamma response (non-responders and responders) to each *Mycobacterium tuberculosis* antigen: **a** ESAT6:CFP10; **b** PstS1/CFP10; and **c** PstS1_(285–374)_:CFP10. *p < 0.05 via Wilcoxon signed rank test. *White square* recent close contact exposed to a multidrug-resistant *Mycobacterium tuberculosis* strain

Despite the presence of a TST positive reaction, some of the rCt submitted to tLTBI lacked baseline IGRA reactivity. We, thus, opted for the stratification of Group III individuals according to their baseline IFN-γ response (responders/non-responders) to each stimulus (Fig. 4). No statistical significant changes were observed in the sub-cohort of non-responder rCt. On the other hand, there was a statistically significant decrease among the LSA-responders to ESAT6:CFP10 (from 758.5 ± 731 to 231.2 ± 240.1 pg/mL, p = 0.027) as well as to PstS1_(285-374)_:CFP10 (from 588.6 ± 650.0 to 106.7 ± 288.4 pg/mL, p = 0.004), albeit with a borderline significance relative to the full PstS1/CFP10 combination (from 469.2 ± 306.8 and 28.0 ± 45.7 pg/mL, p = 0.06). One rCt preventively treated with isoniazid did not show any significant changes in IGRA response modulation over time (Fig. 4, White square). Of note, his/her respective IC was later diagnosed with MDR-*Mycobacterium tuberculosis* (multidrug resistant to isoniazid and rifampicin) infection.

## Discussion

Diagnostic tests to accurately detect an invasive microbe play key roles in patient management and the control of infectious diseases [15]. A previous exploratory investigation conducted by our group showed that, among TB cases, the combination of full PstS1 with CFP10 induced IFN-γ response levels similarly to what occurred following ESAT6:CFP10 stimulation [16]. In another study, differential immune responses to PstS1 peptides were observed [17]. It was also found that the peptide included in our PstS1_(285–374)_:CFP10 fusion protein induced higher T cell proliferative responses in LTBI compared to pulmonary TB donors [17]. Additionally, despite the very low-level expression of the PstS1 protein by *M. bovis* BCG [18], the high immunogenicity of this protein during TB has been proven in a variety of studies [16, 17, 19–22]. In fact, we have likewise reported [13] a similarly high specificity, for LTBI detection, in PstS1_(285–374)_:CFP10 and ESAT6:CFP10 IFN-γ responses (∼90% specificity) [13], including those to the full PstS1/CFP10 (personal communication). Nevertheless, among TST^pos^ contacts, the chimeric protein had the highest detection rate (**WBA**: 23/54, 42.6%; **LSA**: 26/54, 48.2%), followed by ESAT6:CFP10 (**WBA**: 18/54, 33.3%; **LSA**: 24/54, 44.4%) [13], and PstS1/CFP10 (**WBA**: 20/54, 33.3%; **LSA**: 22/54, 40.7%, personal communication). Since the new immunogenic fusion protein PstS1_(285–374)_:CFP10 seems to be more suitable for adding sensitivity to ESAT6:CFP10 than the full PstS1/CPF10 combination, it was decided to investigate further. Indeed, there is much evidence suggesting that IGRA-ESAT6:CFP10 will be false-negative in a significant proportion of both latent and actively-infected *Mtb* individuals [23]. However, in the absence of a gold-standard test for LTBI, questions arose concerning the practical significance of detecting TST^pos^/ESAT6:CFP0^neg^/PstS1_(285–374)_:CFP10^pos^ individuals. Thus, longitudinal studies with individuals recently exposed to *Mtb* should help to clarify any remaining doubts concerning the absolute reliability of the newly-proposed LTBI diagnostic method.

The mechanisms involved in the progression from LTBI to TB are not yet fully understood. The development of TB is associated with a failure in the immune surveillance system of the host with a Th2 polarization that inhibits the host protective Th1 response (IFN-γ, interleukin-12, and tumor necrosis factor [24–27]. The incident cases presented a coincident decrement of WBAs, probably due to an inefficient activation of effector Th1 cell repertories [26]. As seen in Fig. 2e, however, by LSA, memory and resting T cells with a longer incubation period were able to differentiate and initiate IFN-γ production [28–30]. Other studies have shown similar IFN-γ responses in such assays [30–33]. In the end, these occurrences might reveal that some of the host cell populations would still be able to mount a Th1-IFN-γ response to *Mtb* antigens even after the development of TB. It is noteworthy that the small sample size may have limited our ability to detect a significant modulation. As such, it is clear that additional studies are required to validate or not the reported dichotomic IGRA modulation during TB progression.

Individuals from different groups not sharing any independent, mutually exclusive clinical or epidemiological risk factors demonstrated an IGRA conversion. To elucidate these cases, it must be taken into account that: (1) An IGRA response may be affected by a prior TST since the test consists of the intradermal inoculation of a *Mtb* crude antigenic pool [34]; (2) in a high-transmission setting such as Rio de Janeiro, re-exposure to *Mtb* often leads to the maintenance or incensement of the IFN-γ responses [35, 36]; and (3) the lack of a baseline (T_0_) response may be due to the “window period” between exposure to a pathogen and development of an adaptive response. A dynamic characteristic of the IGRA response over time has been previously described [37], Thus, the idea that minor non-specific differences in the IFN-γ response should also be expected. Nonetheless, the reversion to negativity predominantly seen in Group III (Fig. 3) corroborates previous findings regarding LTBI [34, 35, 38–40] and TB [41, 42] individuals receiving antibiotic therapy. Of note is the recent contact of a MDR-TB index case who did not demonstrate a LSA regression post-tLTBI with isoniazid, suggesting that antibiotics were ineffective in this case (Fig. 4, White square). It, therefore, could be surmised that the new LSA PstS1_(285–374)_:CFP10-based might prove useful in monitoring changes in the immune response after completion of preventive therapy.

In the present study, both WBA and LSA displayed a few discordant results. Similar data have been gathered via commercial kits [13, 43]. This result was not surprising since the responses of different immune cell subsets are elicited in short- and long-term cultures [28, 29]. From an operational standpoint, WBA is faster, more cost-effective, and easier. Contrariwise, a significant post-tLTBI regression response during the follow-up period only occurred in LSA (Fig. 3). Therefore, in settings in which higher sensitivity is required, an approach that could be considered would be combining both test results to characterize LTBI [28] since WBA and LSA have particularities that must be reckoned with before application. It should be emphasized that all TB incident cases were detected by WBA-PstS1_(285–374)_:CFP10, LSA-PstS1_(285–374)_:CFP10, and TST, but that the IGRA tests showed better selectivity among the 108 rCt (**WBA-PstS1**_**(285–374)**_ **:CFP10**: 28/108, 25.9%; **LSA-PstS1**_**(285–374)**_ **:CFP10**: 31/108, 28.7%; **TST**: 54/108, 50%) [13]. Moreover, the IGRA PstS1_(285-374)_:CFP10-based showed moderate-to-substantial kappa (κ) agreement under ESAT6:CFP10 stimulation in WBA and LSA (κ = 0.50–0.71), respectively [13]. If these findings are corroborated in further investigations, the use of any of the newly-proposed IGRAs instead of TST may be able to reduce the number of unnecessary tLTBI prescriptions.

## Conclusion

This is the first study to monitor changes in the immune response profile of the IGRA PstS1_(285–374)_:CFP10-based among rCt during a 12-month period. As a result, additional information has been acquired about this new LTBI biomarker in an effort to highlight its potential. Without a doubt, the sample size and prophylactic tLTBI are limitations, which may contribute to an underestimation of the negative/positive predictive values of the test to detect TB progression. However, it is our belief that the participants in our study groups accurately reflect the public at large in routine clinical practice in a scenario with a high TB-burden and BCG vaccination coverage, in which prior *Mtb* exposure or re-exposure after initiating tLTBI cannot be ruled out even with respect to TST^neg^/ESAT6:CFP10^neg^ individuals [2]. Interestingly, except for one outlier with radiological evidence of long-term LTBI, Group I participants (TST^neg^, n = 17) continued having a very low IFN-γ response to PstS1_(285–374)_:CFP10-based WBA and LSA during the entire follow up (Additional file 1: Table S1). LSA regression was predominantly observed among rCt submitted to tLTBI (Group III, PstS1_(285–374)_:CFP10 [n = 09], p < 0.05, Figs. 3 and 4). In addition, both incident cases were detected early by IGRA-PstS1_(285–374)_:CFP10 before showing microbiological evidence of active pulmonary TB. Together, these data provide more positive evidence about the potential of IGRA PstS1_(285–374)_:CFP10-based for LTBI diagnoses. It is of utmost importance that low- and middle-income countries invest in the development of accurate, reliable, and cost-effective LTBI diagnostic tools to ensure the proper maintenance of their TB control programs. In light of the previous [13] and present evidence, we are encouraged to proceed to a next-step: validation of the use of IGRA-PstS1_(285–374)_:CFP10 in cohorts from different geographical areas worldwide.

## Additional file

**Additional file 1: Table S1.** Mean, standard deviation (SD), IFN-g reactivity of whole-blood stimulation.

## Abbreviations

TB: tuberculosis
LTBI: latent tuberculosis infection
tLTBI: treatment for latent tuberculosis infection
rCt: recent close contacts
IFN-γ: interferon-gamma
IGRA: interferon-gamma release assay
WBA: Whole blood short-term assay
LSA: long-term stimulation assay
*Mtb*: *Mycobacterium tuberculosis*
TST: tuberculin skin test
pos: positive
neg: negative
RD1: region of difference 1
Th: T helper
BCG: Bacilli Calmette-Guérin
pg/mL: picograms/milliliter
